# Supplementary material for: HPV-Mediated Resistance to TNF and TRAIL Is Characterized by Global Alterations in Apoptosis Regulatory Factors, Dysregulation of Death Receptors, and Induction of ROS/RNS
Source: Int J Mol Sci. 2019 Jan 8;20(1):198. doi: 10.3390/ijms20010198 (PMC6337392; doi:10.3390/ijms20010198)
Supplement: Supplementary file 1 [file ijms-20-00198-s001.pdf]

Cabeça et al. Figure S1

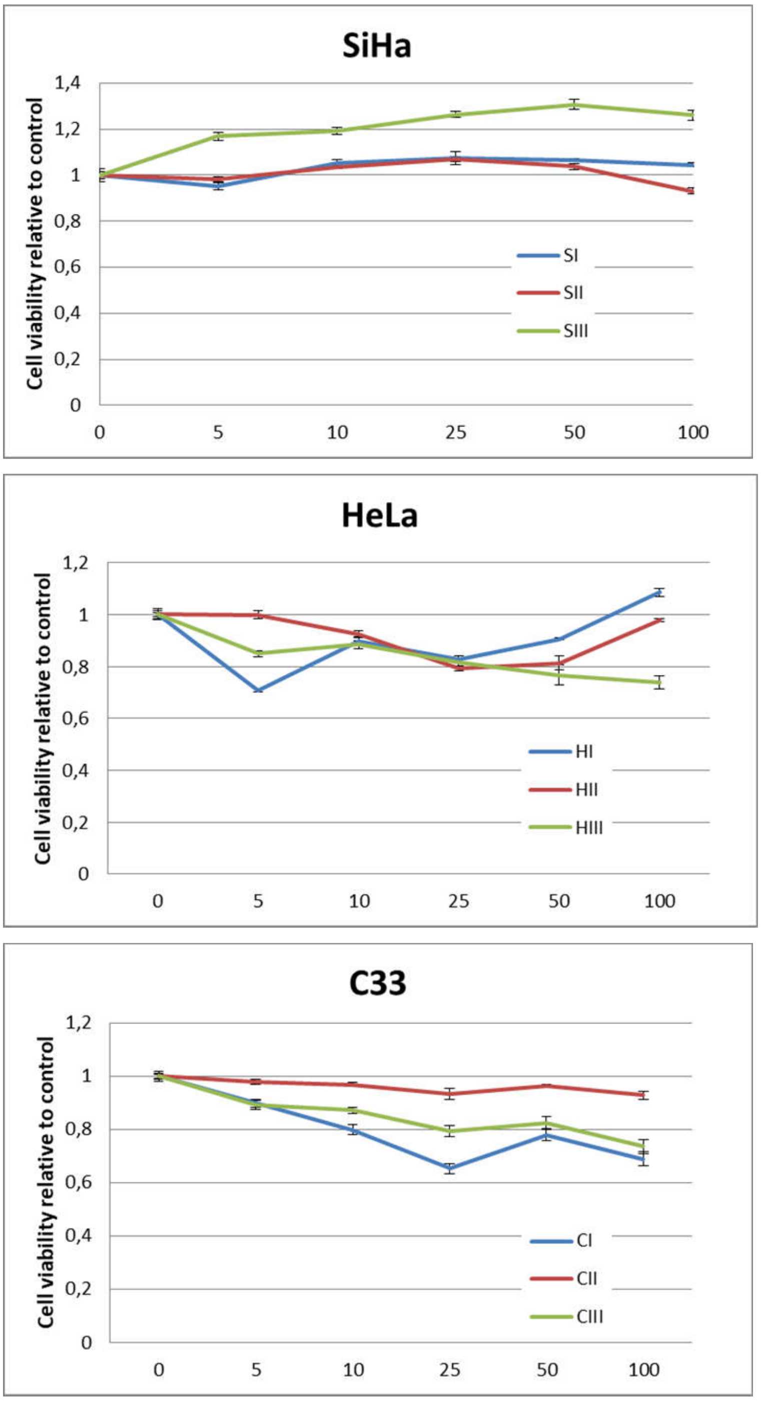

Figure S1 – Cervical cancer derived cell lines are resistant to TRAIL. Effect of TRAIL on cervical cancer derived cell lines SiHa, HeLa and C33. Cells were cultured in 96 wells plates (2000 cells/well) and after 24 hours they were treated with 5, 10, 25, 50 or 100 ng/mL of TRAIL. All treatments were performed in octuplicate. After 60 hours 10 µL of Alamar blue were added per well and cells were incubated at 37° C in an incubator for at least 4 hours. After this period, Alamar Blue's reduction was monitored in a spectrophotometer through absorbance measurement at 570 e 600 nm. The results presented are from three independent experiments (I-III). All data are presented as values relative to control.

Cabeça et al. Figure S2

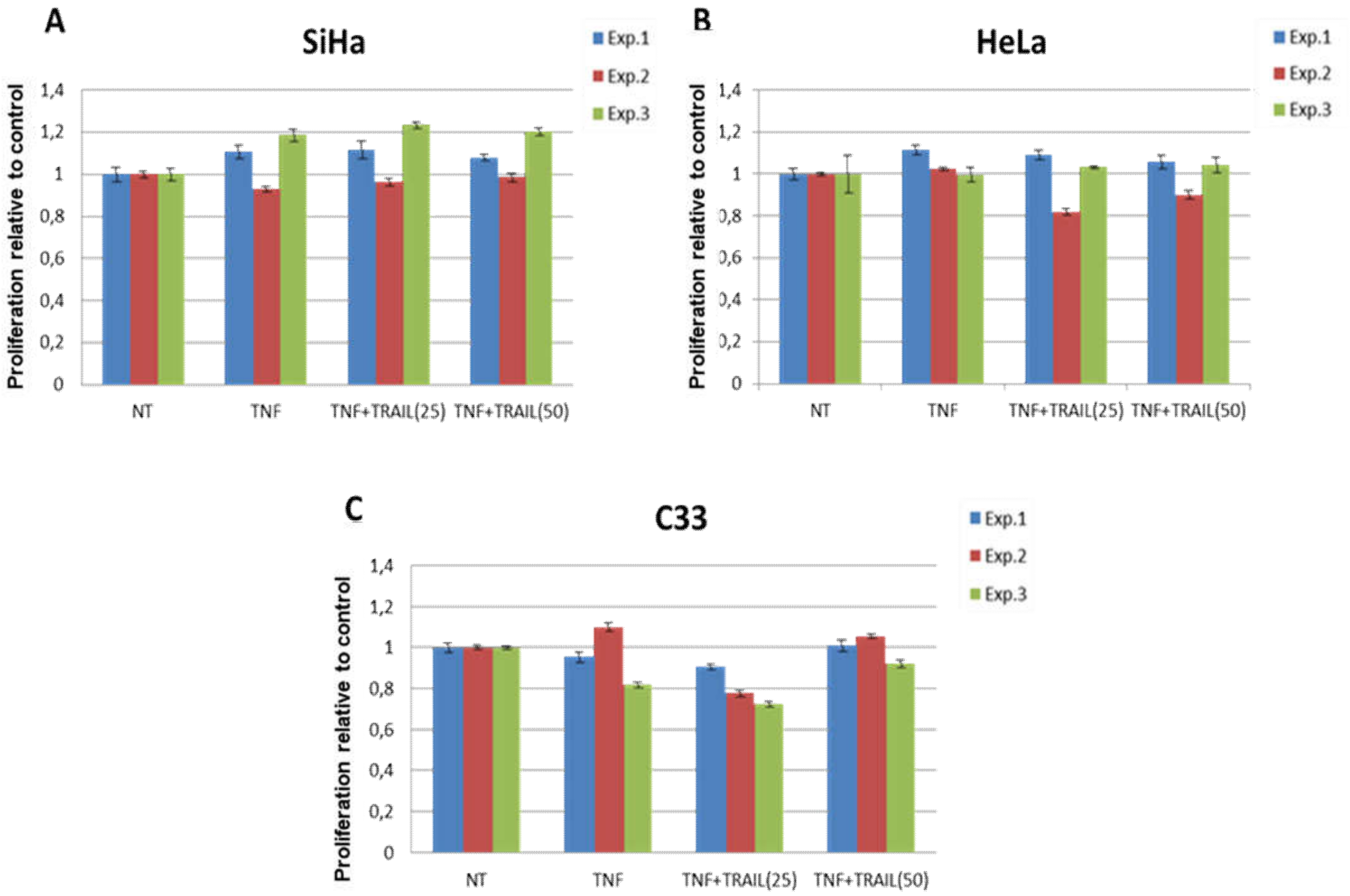

Figure S2 – Cervical cancer derived cell lines are resistant to the combined action of pro-inflammatory cytokines TNF and TRAIL. Cells were cultured in 96 wells plates (2000 cells/well) and after 24 hours they were treated with 2 nM TNF or with 2 nM TNF plus 25 or 50 ng/mL of TRAIL. All treatments were performed in octuplicate. After 60 hours 10 µL of Alamar blue were added per well and cells were incubated at 37° C in an incubator for at least 4 hours. After this period, Alamar Blue's reduction was monitored in a spectrophotometer through absorbance measurement at 570 e 600 nm. The results presented are from three independent experiments. All data are presented as values relative to control.

Cabeça et al. Figure S3

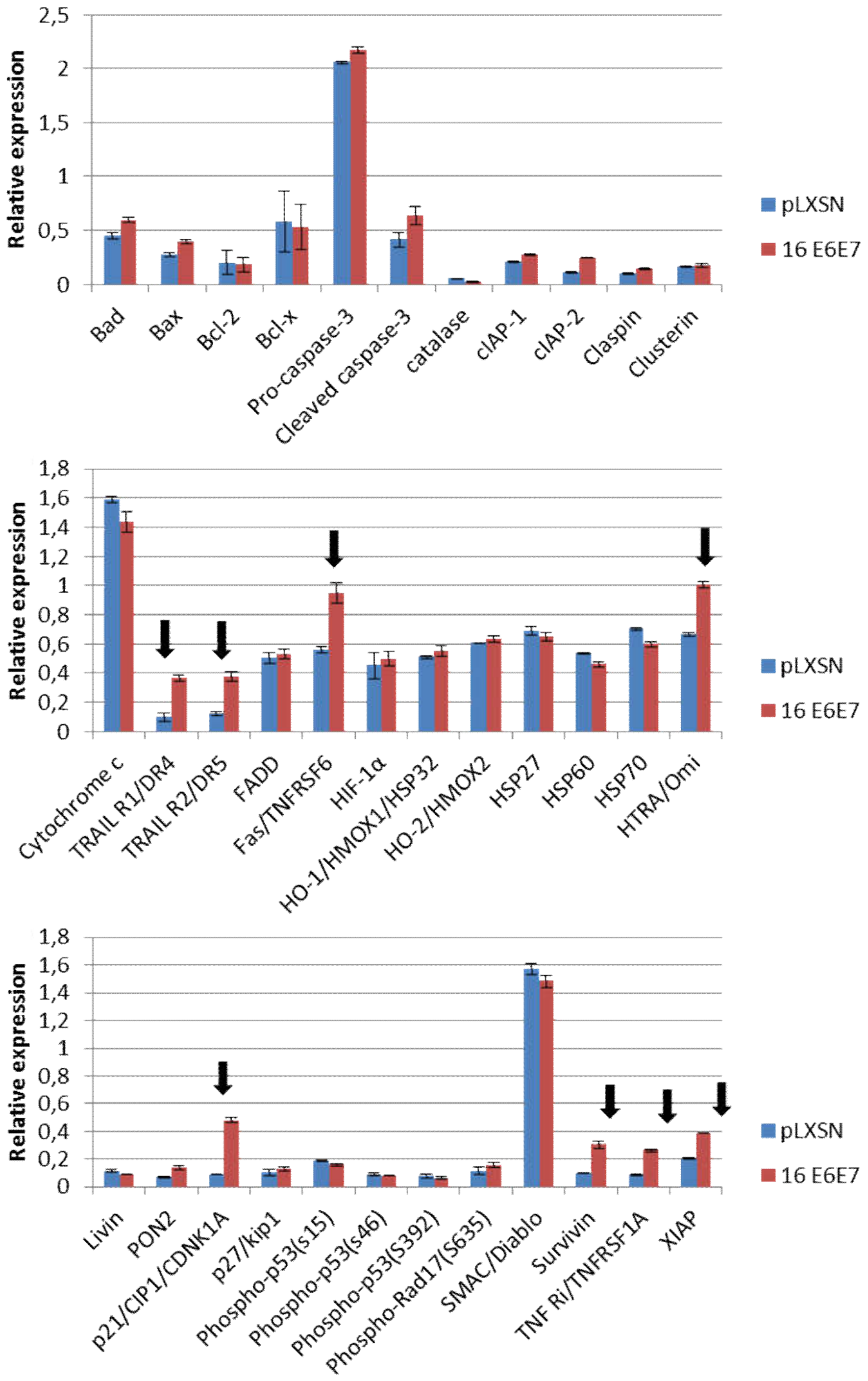

Figure 3 – Effect of HPV16 E6 and E7 in the levels of proteins associated with apoptosis regulation/execution upon treatment with TNF. The expression of 35 proteins involved in the regulation/execution of apoptosis was determined in total protein extracts of epithelial organotypic cultures established from control (pLXSN) and HPV16 E6/E7 transduced PHKs before and after treatment with 2 nM of TNF. For this purpose we used the Proteome ProfilerTM Array - Human Apoptosis Array Kit (R&D Systems, #ARY009), according to the manufacturer's instructions. Signals obtained were quantified using ImageJ software and presented as expression relative to endogenous controls. The results presented are representative of three independent experiments.

Cabeça et al. Figure S4

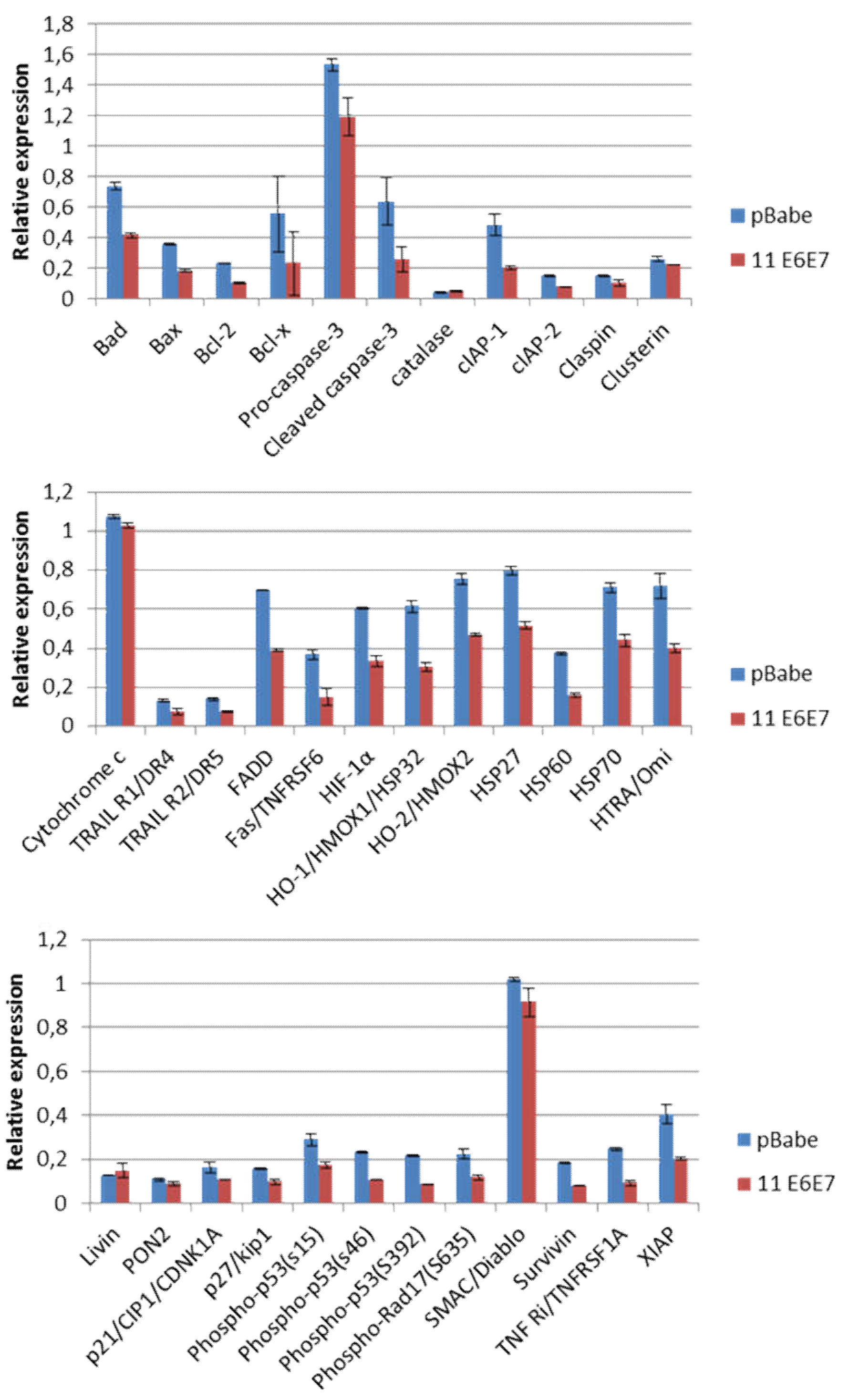

Figure S4 – Effect of HPV11 E6 and E7 in the levels of proteins associated with apoptosis regulation/execution upon treatment with TRAIL. The expression of 35 proteins involved in the regulation/execution of apoptosis was determined in total protein extracts of epithelial organotypic cultures established from control (pBABE) and HPV11 E6/E7 transduced PHKs before and after treatment with 50 ng/mL of TRAIL. For this purpose we used the Proteome ProfilerTM Array - Human Apoptosis Array Kit (R&D Systems, #ARY009), according to the manufacturer's instructions. Signals obtained were quantified using ImageJ software and presented as expression relative to endogenous controls. The results presented are representative of three independent experiments.
